# Laser-Sintering of Cyclic Olefine Copolymer for Low Dielectric Loss Applications

**DOI:** 10.3390/polym16121751

**Published:** 2024-06-20

**Authors:** Manuel Romeis, Michael Ehrngruber, Dietmar Drummer

**Affiliations:** 1 Institute of Polymer Technology, Friedrich-Alexander-University Erlangen-Nurnberg, 91058 Erlangen, Germany; 2 Institute of Microwaves and Photonics, Friedrich-Alexander-University Erlangen-Nurnberg, 91058 Erlangen, Germany

**Keywords:** SLS, selective laser sintering, COC, cyclic olefine copolymer, low dielectric loss, additive manufacturing

## Abstract

With increasing demands for data transfer, the production of components with low dielectric loss is crucial for the development of advanced antennas, which are needed to meet the requirements of next-generation communication technologies. This study investigates the impact of a variation in energy density on the part properties of a low-loss cyclic olefin copolymer (COC) in the SLS process as a way to manufacture complex low-dielectric-loss structures. Through a systematic variation in the laser energy, its impact on the part density, geometric accuracy, surface quality, and dielectric properties of the fabricated parts is assessed. This study demonstrates notable improvements in material handling and the quality of the manufactured parts while also identifying areas for further enhancement, particularly in mitigating thermo-oxidative aging. This research not only underscores the potential of COC in the realm of additive manufacturing but also sets the stage for future studies aimed at optimizing process parameters and enhancing material formulations to overcome current limitations.

## 1. Introduction

The selective laser sintering (SLS) process is a popular technique in the additive manufacturing (AM) space. It offers the possibility to manufacture complex 3-dimensional parts without the need for molds like other processing techniques while still maintaining the needed precision. However, a major limitation of the SLS is its restricted range of materials that can be used in the process. In industrial applications, the materials that are most commonly used are semicrystalline polymers, mainly polyamide 12 (PA12). A specific field where the potential of AM and especially SLS is becoming increasingly relevant is communication technology, particularly for new forms of high-frequency antennas in development for emerging technologies like 6G and 7G networks. While offering geometric freedom, the material limitations of SLS are a significant challenge. The materials that are used in the process do not possess the needed dielectric properties, especially a low dielectric loss, which makes them unsuitable for high-frequency applications. This limitation shows the need for the exploration of new materials that are not only compatible with the SLS process but also have the desired dielectric properties. 

Cyclic olefin copolymers (COC), which are already used for low dielectric loss applications, are a promising candidate to fill this space. Its introduction to the SLS process could potentially overcome the current limitations and open up new possibilities for advanced antenna manufacturing, enabling the SLS process to emerge into the field of high-frequency applications. For this reason, research and development are needed in order to integrate COC and potentially other low-loss materials into the SLS process. This study presents the generation and rheological optimization of COC powder from granulate by grinding for processing in the SLS process. The manufactured powder was subsequently processed in the SLS process with varying energy densities and analyzed according to their density, surface quality, thermal aging, dielectric properties, relative permittivity, and dielectric loss, respectively, as these factors are critical for an application as antennas in high-frequency technology.

## 2. State of the Art

In the SLS process, a high-energy laser irradiates and, due to the introduced energy, melts a polymer powder to form a solid 3-dimensional structure, which is based on a digital design without the need for a mold or tool. This process starts with a roller or blade uniformly distributing the polymer powder over a heated build platform at a defined thickness. This layer is subsequently locally irradiated with the laser in the shape of the cross-section of the parts, and therefore, the polymer particles fuse to each other and to the already-built layer underneath. The powder bed is then lowered by one layer height, and the process is repeated until the complete part is generated [1,2]. During this process, the surrounding powder forms a compact powder cake and stabilizes the part. Therefore, no support structures are needed like in other AM techniques, such as fused filament fabrication (FFF) or stereolithography (SLA) [3]. 

In the field of SLS, semicrystalline polymers are far more researched and utilized in applications due to their increased mechanical performance compared to amorphous polymers, mostly in the form of PA12 or PA11 [4,5,6,7]. Due to their increased part densities, these semicrystalline polymers typically show enhanced strength and durability compared to the amorphous ones, which makes them favorable for a wide range of practical applications. However, there has also been notable research activity with amorphous polymers for SLS. The most important and researched among these are ABS [8], polystyrene [9,10], polycarbonate [11,12], and PMMA [13,14]. Due to their unique material properties, they are preferred in specific applications, despite their drawbacks in density and mechanical strength. The main differences between semicrystalline and amorphous polymers in SLS are mostly their final part density, mechanical properties, and dimensional accuracy. While semicrystalline polymers generally offer higher part densities and, therefore, increased mechanical properties, amorphous polymers normally show a higher dimensional accuracy, which can be an important factor for precision-focused parts in engineering and manufacturing applications [15]. 

Additive manufacturing is increasingly used in the manufacturing of high-frequency (HF) parts, such as waveguides. In the most prominent method, a polymer base body is fabricated in an AM process and subsequently metalized [16,17,18]. Conventionally, this metallization is mostly performed on the inside of the waveguides, which comes with the drawback that the inside of the part has to be accessible. A new innovative development uses metallization on the outside of the parts, which offers increasing flexibility and freedom in the part design since there is no need for accessibility to the inside of the part combined with a potential reduction in size and, therefore, better integration possibilities within the final application of the HF component. This technique also allows post-treatment of the critical surfaces where the metallization is applied, which allows a higher surface roughness during the manufacturing of the part. However, since the electrical field interacts in the case of the outside metallization with the polymer, this technique needs polymers with a low dielectric loss for the creation of efficient HF components. Continued research and development are essential to refining the practicality and efficiency of this method. Regarding suitable materials for waveguide antennas, low-loss plastics like PTFE, PEI, PP, PE (particularly those with high molecular weights), PEEK, and COC [19] are the most promising. Nonetheless, these materials present certain challenges for additive manufacturing owing to their unique melting and sometimes crystallizing properties. Of these options, COC is among the minimal dielectric losses.

The most important factor for the dielectric properties of polymers is the chemical structure of the material itself, which leads to different polarization effects [20]. When under the influence of an alternating external electrical field, different dielectric responses emerge depending on the frequency of the field. Interfacial polarization, being the slowest response in the area up to 100 Hz, can cause very high losses but is not to be expected in high-frequency applications and is more present in sole dielectric materials or capacitors. In polymers, the expected dielectric responses are, with increasing frequency, dipolar (< 10^8^ Hz), atomic (<10^13^ Hz), and electronic (<10^15^ Hz) polarization effects [21,22]. Consequently, for the frequencies studied in this research of 10–50 GHz, mainly atomic polarization effects can be expected while still being close to the range of dipolar polarization effects and, therefore, influencing the overall dielectric properties of the samples. Besides the internal material influence, external factors such as the temperature [23] and the moisture content [24] can impact the dielectric properties.

## 3. Materials and Methods

### 3.1. Material

**Figure 1:**
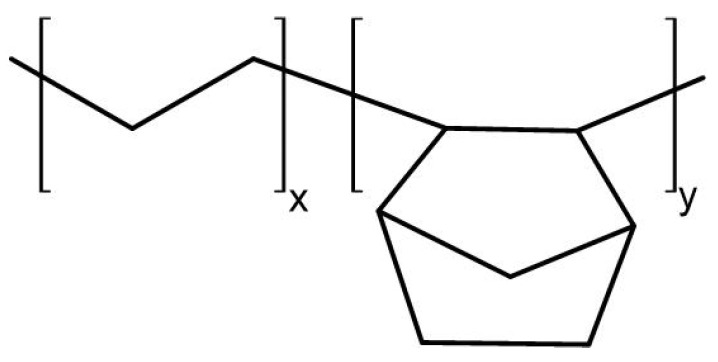
Chemical structure of COC.

For the processing of COC in the SLS process, a type of Topas COC-Blend 16-024 granulate by Polyplastics Co., Ltd. (Tokyo, Japan) was purchased and used. The typical chemical structure of COC is shown in Figure 1. 

Prior to the SLS process, the material had to be milled with a ZM 300 impact mill by Retsch GmbH (Haan, Germany) with the usage of an 80 µm sieve. The granulate was heavily cooled prior to milling in liquid nitrogen to reduce impact strength and, therefore, improve particle breaking. The milled powder was further sieved through a 150 µm sieve with a vibrational sieve tower to ensure no bigger particles were included. To enhance the flowability, the powder was mixed with 0.6 wt.% fumed silica aerosil type R 972 by Evonik (Marl, Germany) with a paint can mixer at a speed of 60 rpm for 15 min. The used aerosil has an average particle size of 16 µm and a specific surface of 90–130 m^2^/g. Due to the nonpolar structure and the low amount added, no significant impact on the dielectrical properties of the powder is expected.

### 3.2. Part Generation

Part manufacturing was performed on a P396 by EOS GmbH (Krailing, Germany) under a nitrogen atmosphere with a modified smaller building chamber with a xy-size of 100 × 100 mm^2^. The used parameters can be seen in Table 1. The area of the parameter room was developed in pre-trials based on a full-factorial investigation of single layers. 

It has to be noted that the build-chamber temperature is slightly above the measured glass transition temperature since temperatures below 180 °C still show warping of the manufactured parts. The literature shows that for amorphous polymers, build chamber temperatures close to T_g_ are suitable for processing. Eight different exposure settings from 10 W to 24 W were used in the experiments with varied laser intensity. With the formula for the energy density

$$\mathrm{energy}\;\mathrm{density}=\frac{\mathrm{laser}\;\mathrm{power}}{\mathrm{hatch}\;\mathrm{distance}\;\times \mathrm{scan}\;\mathrm{speed}\;\times \mathrm{layer}\;\mathrm{height}}$$

The values presented in Table 2 are calculated.

The parts were irradiated in ascending order according to the laser power. The testing samples were blocks with dimensions of 10 × 5 × 25 mm^3^. Subsequent to manufacturing, the samples have been machined to exactly the size of 21.36 × 6.71 × 2.37 mm^3^, predetermined by the chamber size of the resonator for dielectric measurement. Machining is necessary to ensure perfect contact with the resonator chamber since gaps would heavily affect the measurement.

### 3.3. Methods

#### 3.3.1. Material Analysis

In order to choose a suitable processing temperature, a comprehensive thermal analysis of the COC material was performed. This analysis involved differential scanning calorimetry (DSC) and thermogravimetric analysis (TGA). DSC was used to determine the glass transition temperature (T_g_), which is critical for setting the optimal temperature of the print chamber. If the temperature is set too low, no melting and, therefore, no part generation can be achieved. Also, due to deviations in cooling of the prior layer and the irradiated layer and, therefore, shrinkage, warpage can occur. If the processing temperature is set too high, the powder can melt without the interaction of the laser, and the recoating process can be negatively impacted. TGA was conducted to determine the maximum temperature that the powder can withstand inside the heated build chamber without thermal degradation. In combination, these analyses provide a detailed understanding of the thermal properties of the powder, enabling an optimal processing temperature for further processing in SLS. DSC was performed according to DIN EN ISO 11357-1 [25] and TGA according to DIN EN ISO 11358 [26], both on a Jupiter STA 449 F3 by Netzsch (Böblingen, Germany). As the heating rate, 10 K/min was chosen. DSC was performed under a nitrogen atmosphere, and TGA was performed under a nitrogen and oxygen atmosphere, respectively. 

For powder characterization, SEM was performed on the virgin, non-modified, but milled and sieved powder. The used SEM was a Gemini Ultra-Plus by Carl-Zeiss AG (Oberkochen, Germany) with 5 kV and various magnifications. The measurement of particle size distribution was conducted with the virgin powder in dry state on a Morphologi G3 by Malvern Panalytical (Malvern, UK) with a magnification of five. Both the numerical and volumetric particle sizes have been analyzed. The compression depth measurement developed by Hesse [27] was conducted on an HR-2 rheometer by TA Instruments (New Castle, DE, USA) with a 25 mm plate and the matching cylinder. The powder was hand-sieved in a dry state into the cylinder and smoothed with a scraper at a 45° angle. Afterward, the upper plate was axially moved until a pre-force of 0.2 N was achieved to ensure constant starting conditions for each measurement. After reaching the pre-force, the powder was compressed with an axial speed of 1 µm/s until an axial force of 5 N was reached. Bulk density was determined according to DIN EN ISO 60 [28] on an ADP measurement tool by KARG Industrietechnik (Krailing, Germany). The powder was tested for moisture content by Karl Fischer titration with a VA-200 vaporizer and CA-200 moisturemeter by A1 Envirosciences (Düsseldorf, Germany). 

#### 3.3.2. Part Analysis

**Figure 2:**
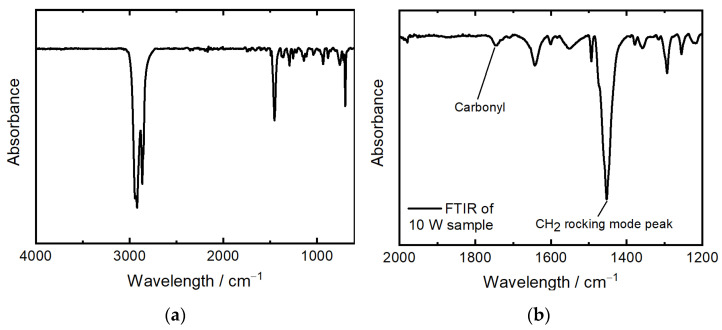
ATR spectrum of (**a**) COC granulate and (**b**) COC samples manufactured at 10 W in the SLS process.

Prior to the part analysis, the parts were blasted with glass beads to remove adherent powder. The manufactured parts have been analyzed according to their density, surface roughness, geometric accuracy, and dielectric properties. The density of the powder was measured according to DIN EN ISO 1183-3 [29] with an Accu Pyc 1330 Gaspycnometer by Micromeritics GmbH (Unterschleißheim, Germany) at 25.4 °C with helium gas in order to receive the density of the material. The part density is calculated through the ratio of the mass and the volume, while mass is measured with a precision scale. The part dimensions were measured at three points, each with a micrometer screw in all three directions. The surface roughness was analyzed with a laser-scanning microscope by Keyence (Osaka, Japan) at a magnification of 10. The top and side surfaces, according to their positioning in the build chamber, have been investigated. For the determination of thermo-oxidative aging, all samples have been investigated via ATR-FTIR in reflection on a Nicolet 6700 and Microscope Continuum by Thermo Scientific (Waltham, MA, USA). The spectra have been measured with 16 scans and a resolution of 2 cm^−1^ at the full spectrum in between 4000 cm^−1^ and 600 cm^−1^. See Figure 2a for the spectrum of the samples manufactured at 10 W.

The measurement of thermal aging was conducted via the analysis of the carbonyl index (CI), a well-established method for the quantification of oxidative degradation in polymers. The carbonyl index is calculated with the ratio of absorbance at two specific wavenumbers: the carbonyl absorption peak around 1750 cm^−1^, which resembles the presence of carbonyl oxidation by-products that are typically formed during the thermal aging of COC, and the CH_2_ bending vibration peak at 1456 cm^−1^, which is used as a reference peak (see Figure 2b). The formula used for the CI (see Equation (1)) calculation uses these absorbance values, adjusted for baseline.

(1) $$\mathrm{CI}=\frac{\mathrm{Absorption}\;\mathrm{at}\;\mathrm{maximum}\;\mathrm{of}\;\mathrm{carbonyl}\;\mathrm{peak}\;\left(1750{\;\mathrm{cm}}^{-1}\right)}{\mathrm{Absorption}\;\mathrm{at}\;\mathrm{internal}\;\mathrm{thickness}\;\mathrm{band}\;\left(1456{\;\mathrm{cm}}^{-1}\right)}$$

The baseline method makes sure that the calculated CI only considers the changes that are affected by thermal aging, therefore providing a reliable measure of the material’s degradation over time [30,31]. The dielectric characterization, relative permittivity, and loss tangent, respectively, were performed using a specially designed rectangular cavity resonator in connection with a Keysight PNA-X N5247B Network Analyzer (Keysight, Santa Rosa, CA, USA). For this measurement, the test samples must be precisely cut to fit the dimensions of the cavity of the resonator, which are 6.71 mm × 2.37 mm × 21.36 mm, ensuring they perfectly fill the cavity. The resonator was configured to allow for the evaluation of at least eight resonances within the measurement range of 5 GHz to 67 GHz. The permittivity was determined based on the resonance peaks that formed in the graph. This involved identifying the resonance frequency of the sample by identifying its resonance peaks and comparing these with the resonance peaks from a baseline measurement in the empty resonator cavity. The permittivity was then calculated based on the frequency shift between these two sets of data. The dielectric loss tangent was determined using the unloaded quality factor of the weakly coupled resonator, identified through the resonance peaks. This quality factor was calculated from the bandwidth at the half maximum of the resonance peaks while using all measurement points around a resonance frequency until the value at the resonance peak was reduced to half. Comparisons were again made between the resonances in the baseline measurement and those obtained with a dielectric material in the cavity. The measurements were performed at room temperature. 

## 4. Results

### 4.1. Material and Particle Properties

**Figure 3:**
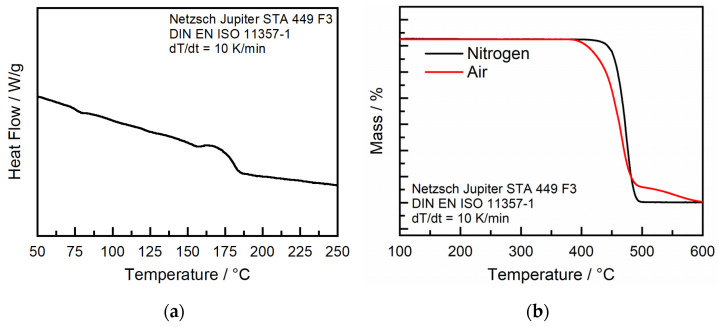
(**a**) DSC and TGA (**b**) of Topaz COC Blend 16-024.

The DSC analysis (Figure 3a) shows a glass transition temperature (T_g_) of approximately 176 °C for the investigated COC. In the additive manufacturing processes of amorphous thermoplastics, it is important to set the build chamber temperature as close as possible to the identified T_g_ to receive the most optimal processing conditions. The reason for this is, firstly, to prevent warping of the material, which can occur due to deviating cooling and, therefore, shrinking processes in and in between the layers, and secondly, to enable proper melting of particles to generate coherent and mechanically resilient structures. Processing near T_g_ allows the particles to fuse effectively while maintaining the structural integrity of the surrounding powder. Excessive temperatures in the build chamber that exceed T_g_ can lead to unintended adhesion of the particles to each other, which can interfere with the flowability of the powder and, therefore, lead to problems with the recoating process or, in extreme cases, the melting of the polymer powder without the interaction of the laser.

**Figure 4:**
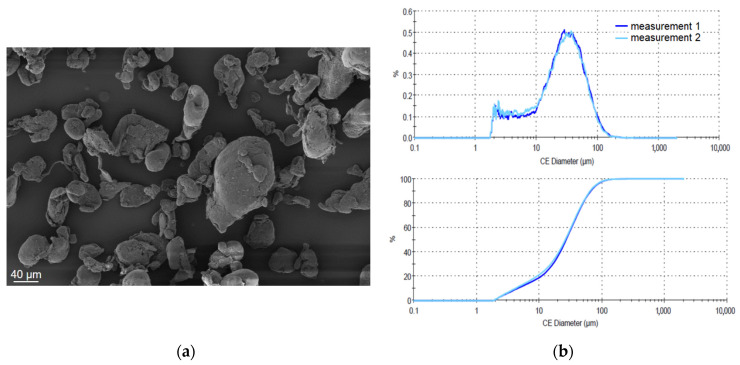
(**a**) SEM of particles and (**b**) particle distribution.

The TGA measurement shows thermal stability until 410 °C in the nitrogen atmosphere and 380 °C under the air atmosphere, respectively, so the used build chamber temperature is in no critical area regarding thermal degradation. TGA has been conducted for both gases to include the case for remaining oxygen in the build chamber. Under oxygen, the thermal degradation starts earlier but is still above the temperatures that are expected during the SLS process. However, it is important to note that COC tends to undergo thermo-oxidative aging, which needs to be further investigated in the FTIR measurements. Figure 4a shows the morphology of the powder particles after milling and sieving. The reviewed particles show a relatively round shape, which should improve the flowability. The quantitative particle distribution measurement shows the peak at about 40 µm, while only a small quantity is above 100 µm, so processing should be possible.

**Figure 5:**
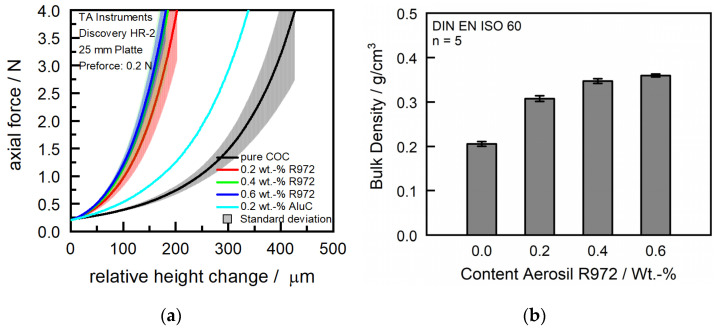
(**a**) Compression depth and (**b**) bulk density.

Besides the particle size, the rheological properties are a crucial part of the SLS process. Figure 5 shows the compression depths, introduced first by Hesse [27], an analysis tool to determine the flowability of a powder that is more reliant than classical rheological analysis like bulk density or Hausner factor. Figure 5a shows the compression depths for the pure and modified COC powders. For a tested commercial PA12 powder, a compression depth of approximately 90 µm is common, but pretests with experimental powders showed that a compression depth of less than 180 µm led to an evenly flow and recoating during processing. 

The analyzed virgin COC exhibits comparatively high compression depth and bulk density. However, these properties were not sufficient for a reliable and uniform recoating process, a hypothesis that was confirmed during coating tests. In contrast, PA2200 demonstrated a compression depth of about 95 µm and a specific bulk density of 0.45 g/cm^3^ [32], with preliminary studies indicating that materials with compression depths lower than 200 µm are generally more suitable for processing. To improve the flow characteristics of the COC powder, the study experimented with adding aluminum oxide type AluC and fumed silica type Aerosil R 972 as flow-enhancing additives, both by Evonik. However, AluC did not yield a significant improvement in the compression depths of COC, but Aerosil significantly decreased compression depth and increased bulk density, as seen in Figure 5. Further attempts to modify the flow properties of COC involved increasing the concentration of aerosil from 0.2% to 0.6%. This modification resulted in only a marginal increase in compression depths and bulk density, reaching a plateau that suggested limited efficacy beyond this concentration. The Karl Fischer titration measured a moisture content of 153 ppm for the milled and modified powders.

Through a modification of the particle shape, e.g., rounding, further improvements in the rheological behavior could be made. It is important to consider that the increased flowability could also lead to insufficient stability of the part cake in the building chamber. Excessive flowability might lead to reduced cohesion in the powder bed, a factor that is crucial for the quality and consistency of the part cake and, therefore, the integrity of the parts inside. This balance between flowability and cohesion remains a key factor in optimizing the properties of materials like the COC used for additive manufacturing applications [33,34].

### 4.2. Part Properties

**Figure 6:**
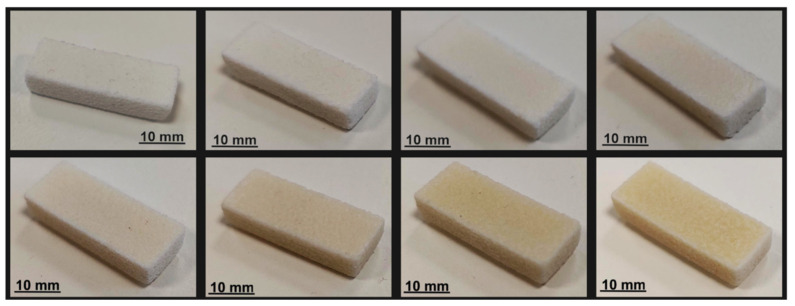
Manufactured COC parts; line one: 10 W, 12 W, 14 W, 16 W (from **left** to **right**); line 2: 18 W, 20 W, 22 W, 24 W (from **left** to **right**).

Figure 6 shows the manufactured parts dependent on the energy density. A direct relationship is shown between the increase in laser power, and consequently energy density, and the discoloration (yellowing) of the manufactured parts. This phenomenon is probably caused by the increased energy induced by the polymer during the laser irradiation. These increased energy levels could possibly lead to enhanced thermo-oxidative stress on the material. This effect is consistent with the behavior seen in the processing of COC and is a known occurrence in standard polymer processing techniques [30]. To verify this effect, an ATR analysis is performed. This is important since an impact on the dielectric properties due to aging has not been investigated. COC studies have shown this effect for olefine polymers and polyethylene, respectively [35,36], due to the formation of polar degradation products. 

**Figure 7:**
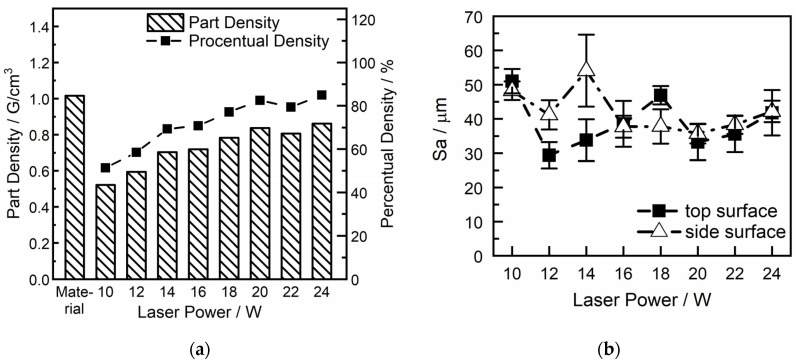
Part density (**a**) and surface roughness (**b**) of LS-COC parts.

The part density of the measured parts depicted in Figure 7 shows a relative density of up to approximately 80% at peak, which is rather low but quite common for amorphous materials processed in the SLS process due to the sintering instead of melting of the particles.

The course of the graph indicates a direct correlation where part density increases with an increase in energy density, presumably due to better melting and, therefore, consolidation of the particles [37]. The literature shows that this trend normally continues until a certain threshold, beyond which a plateau in part density is observed at high energy densities. The reason for this effect is due to an equilibrium between better consolidation and degradation of the polymer due to excessive temperature [36]. During experimentation, an increase in laser intensity was halted at a 24 W threshold due to visible fuming observed during irradiation, indicating possible thermal degradation or material sublimation at higher energy inputs. Despite the observed plateau, it is expected that further optimization of scanning parameters, such as laser speed, hatch spacing, and pattern, could potentially still enhance part density beyond the current observations. As stated, the literature [36] on this topic supports the observation that part density increases with energy density up to a certain point, after which further increases in energy density lead to a decrease in density due to material degradation and the formation of gases during the melting. For better accuracy, the density was also measured via the buoyancy method, but due to the low density at low laser power, no sufficient results could be received since the test medium penetrated these parts. This suggests that the plateau reached may not represent an absolute limit but rather a point of optimization within the current set of parameters. 

With commercial PA12 having a mean surface roughness of 14 µm, the measured values for the COC in Figure 7b are near a desirable range, although the values show a high standard deviation. This could be optimized with a more advanced powder grinding process. Although sieving was conducted on the powder, there are still some bigger than desired particles in the powder, which can be seen in the SEM pictures in Figure 4, which impact the surface values. However, since the particles do not fully melt in the SLS process of amorphous polymers, higher surface roughness compared to semicrystalline polymers can be expected. 

**Figure 8:**
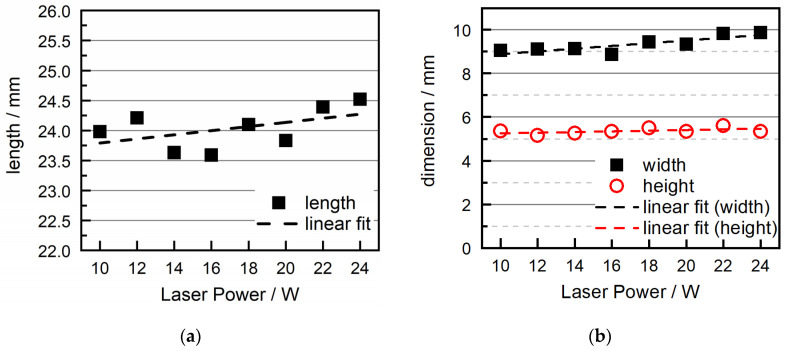
Dimensional accuracy: (**a**) length and (**b**) width/height of SLS-COC parts in dependence on laser power.

Figure 8 shows the measurements for dimensional accuracy. While still having quite some deviation, the measurements for the length of the samples show a linear trend for higher length with increasing laser power.

The samples’ heights depicted in Figure 8b show a mostly stable value slightly above the target value of 5 mm, with a minor increase with higher energy densities. This is a typical effect for the SLS process since the absorption depths of the powder to the laser surpass one layer height, which leads to higher layer heights for the first layer [38]. This deviation can be improved with the adjustment of the irradiation settings for the first layer.

**Figure 9:**
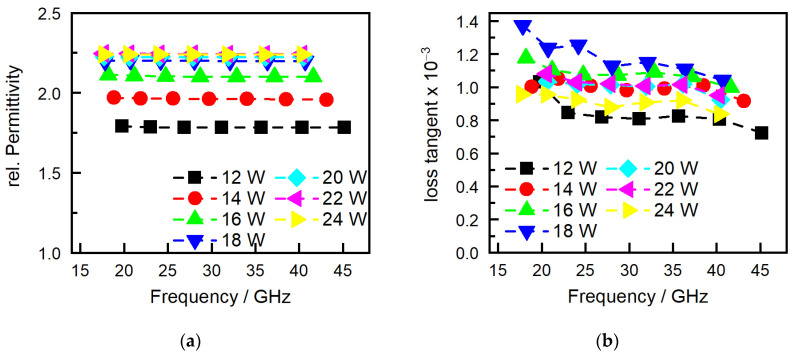
(**a**) Relative permittivity and (**b**) dielectric loss tangent for different frequencies and laser intensities.

Figure 9 shows the measurements of the relative permittivity and loss tangent. The observed increase in permittivity correlates directly with measurements of the part density, as seen in Figure 7a. As laser power increases, the density of the material also increases, which, in turn, enhances the relative permittivity. This relationship can be attributed to the more compact material structure and reduced porosity achieved at higher energy densities. Since the permittivity of air is approximately 1, any increase in material density effectively reduces the amount of air in the part’s microstructure, thereby increasing the material’s overall permittivity. This effect has already been demonstrated in fused filament fabrication (FFF) with the controlled variation in infill percentages [39]. This infill percentage directly impacts the density of the internal structure of the manufactured FFF part and, therefore, part properties like mechanical strength. The said study has also shown that as the infill percentage increases, resulting in higher density, there is a corresponding increase in relative permittivity. This outcome was consistent across different infill percentages, which transferred to the SLS process, which explains the impact of laser-induced density on the electrical properties of materials. Initially, at low energy densities, there is a clear correlation between the loss tangent and the material’s density. As the energy density increases, resulting in a denser material, the loss tangent similarly increases. However, as the energy density continues to rise beyond a certain threshold, this direct correlation with the loss tangent ceases to be consistent. At high energy densities, the behavior of the loss tangent becomes erratic, showing no consistent change and eventually decreasing with further increases in energy density. While the relative permittivity is constant over the measured frequencies, the loss tangent shows a decline with increasing frequency for all samples.

**Figure 10:**
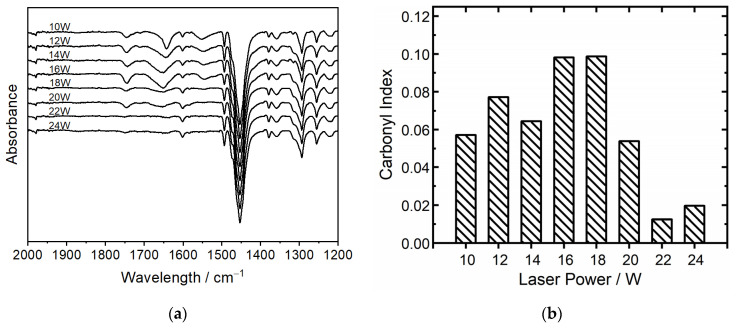
(**a**) ATR measurements are dependent on laser intensities and (**b**) carbonyl index at 1750 cm^−1^.

**Figure 11:**
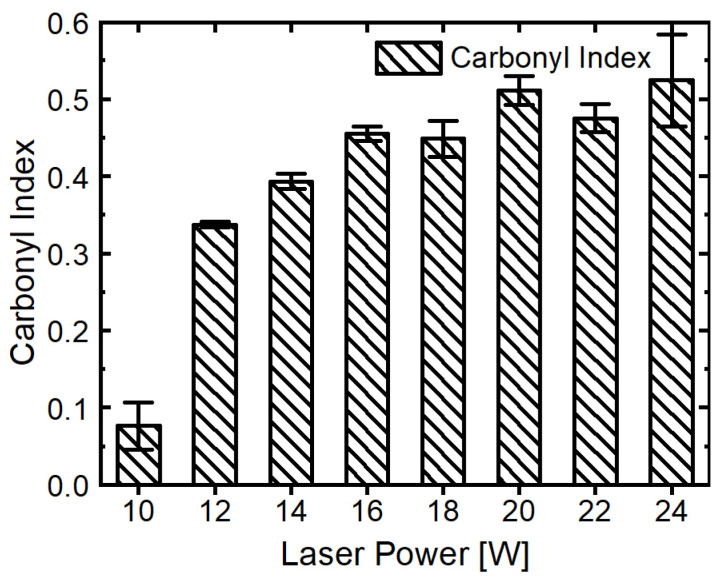
Carbonyl index of single layers at 1750 cm^−1^.

The decrease in the loss tangent at higher energy densities is paralleled by the behavior of the carbonyl index (CI), shown in Figure 10b, which measures the concentration of carbonyl groups, indicative of oxidative degradation at 1750 cm^−1^ in the presented ATR spectra in Figure 10a. With carbonyl groups being highly polar, this can significantly influence the loss of tangent. The observed trends in the loss tangent and carbonyl index with varying energy densities can, therefore, probably be explained through the interaction between material density and thermo-oxidative aging. The thermo-oxidative aging process is known to be not solely dependent on temperature but also on the duration of exposure [40]. The manufactured parts are scanned sequentially with increasing laser power, which means that parts subjected to lower power (e.g., 10 W) are exposed for longer durations to high temperatures compared to those processed at higher power (e.g., 24 W). This change in exposure time can lead to variations in thermo-oxidative aging, subsequently influencing the carbonyl index and, by extension, the loss tangent of the material. Overall, all measured dielectric properties are mostly in the target area for the antenna application of a maximum of 10^−3^ at the target frequency of 40 GHz. To see the changes in aging without any machine- or scanning-related influences, three single layers of each laser intensity were produced, all in individual build jobs. These were irradiated and all exposed to the atmosphere of the building chamber without recoating for 10 s. These single layers have also been analyzed in terms of the carbonyl index in dependence on the laser power (see Figure 11).

These results show the expected increase in aging with increasing laser intensity, confirming, when kept at constant exposition to the heated build chamber, the impact of temperature and time on the aging of COC. Since layer times and, therefore, the exposition to the atmosphere of the build-chamber in the molten state vary depending on the cross-section and number of parts in the SLS process, the prevention of thermal aging is an important topic for future research. Although the dielectric loss is overall at a low level, thermal stabilization has to be conducted on the COC for the SLS process to receive consistent part properties independent of the irradiation sequence. 

## 5. Conclusions

In the presented study, a low-dielectric loss cyclic olefin copolymer (COC) was successfully milled into a defined powder, rheologically modified, and subsequently processed in the SLS process. With the addition of fumed silica, the flowability of the powder could be improved in a defined way so that the recoating process was stable and uniform. To achieve an optimal processing process, the glass transition temperature was identified via thermal analysis, and a chamber temperature adjusted slightly above the glass transition temperature was found to be sufficient for optimal part generation. Under the set processing conditions, parts with varying laser intensities from 10 to 24 W were manufactured. It could be seen that an increase in laser power led to higher part densities as well as better precision in the dimensional accuracy of the parts. However, this also led to an increase in the yellowing of the parts, probably an effect of thermo-oxidative aging, which is a common occurrence with non-stabilized COC materials processed at elevated temperatures. This effect could later be confirmed in the analysis of the carbonyl index of the manufactured parts, which increased with the duration of exposure to the elevated temperatures in the building chamber. An impact of the variation in the laser intensity on thermo-oxidative aging could not be seen in the measurements. Although the increase in energy density resulted in higher part densities, for the SLS process, a typical plateau in density could not be seen. The parts attained an approximate 80% relative density for the highest intensities used in this study, which is a common area for the SLS of amorphous polymers. The increase in laser power also showed an impact on the dielectric properties, specifically causing an increase in relative permittivity due to reduced part porosity and, consequently, a lower ratio of air within the part. This effect could also be seen for the loss of tangent at low laser powers but was inconsistent for high laser powers. This deviation was probably caused by the superstition of the changes in air ratio in the parts and the thermo-oxidative aging during the exposition to the chamber temperatures. Although further optimization of the process parameters and potential stabilization against thermo-oxidative aging could enhance the dielectric properties, this study already underscores the significant potential of laser sintering COC for the additive manufacturing of components with low dielectric loss.

## Figures and Tables

**Table 1:** Process parameters.

Parameter	Value
T_Buildchamber_	180 °C
T_Chamber_	100 °C
h_Layer_	120 µm
d_Hatch_	0.2 mm
v_Scan_	1000 mm/s
v_Recoating_	100 mm/s
V_Recoater_	100 mm/s

**Table 2:** Energy Density.

Sample Number	Energy Density [J/mm^3^]
1	0.42
2	0.5
3	0.58
4	0.67
5	0.75
6	0.83
7	0.92
8	1

## Data Availability

The data presented in this study are not publicly available due to ongoing research in this field.
